# Biodegradable polymeric nanoparticles increase risk of cardiovascular diseases by inducing endothelium dysfunction and inflammation

**DOI:** 10.1186/s12951-023-01808-3

**Published:** 2023-02-24

**Authors:** Wen Shi, Atik Rohmana Maftuhatul Fuad, Yanhong Li, Yang Wang, Junyang Huang, Ruolin Du, Guixue Wang, Yazhou Wang, Tieying Yin

**Affiliations:** 1 Key Laboratory for Biorheological Science and Technology of Ministry of Education, State and Local Joint Engineering Laboratory for Vascular Implants, Bioengineering College of Chongqing University, Chongqing, 400044 China; 2 School of Medicine, Chongqing University, Chongqing, 400030 China

**Keywords:** Biosafety, Polymer, Biodegradable nanoparticles, Cardiovascular diseases

## Abstract

Biodegradable polymers are expected to be an alternative to plastics. Because of its high biocompatibility, poly (lactic-co-glycolic acid) (PLGA) is widely used in medicine. It has been reported that micro-nano plastics can be accumulated in the circulatory system and cause tissue injury. With the increasing environmental exposure of degradable polymer nanoparticles (NPs), the impact of this risk factor on cardiovascular disease deserves attention. Thus, we aim to study the harmful effect of PLGA NPs on the process of vascular stenosis which is a typical pathological feature of cardiovascular diseases. We establish a mouse vascular stenosis model with intravenously injecting of PLGA NPs for 2 weeks. This model leads to a significant narrowing of the left common carotid artery which is characterized by the increasing intima area and focal stenosis. We observe that PLGA NPs accelerate stenosis progression by inducing inflammation and impairing vascular function. It promotes the proliferation of smooth muscle cells and causes abnormal collagen distribution. The combination of wall shear stress and PLGA NPs uptake speed up endothelial cell damage, decrease endothelial permeability and cell migration capacity. Our results suggest that PLGA NPs may pose a risk in cardiovascular stenosis which inspire us to concern the biodegradable polymeric materials in our living especially the clinic applications.

**Graphical Abstract:**

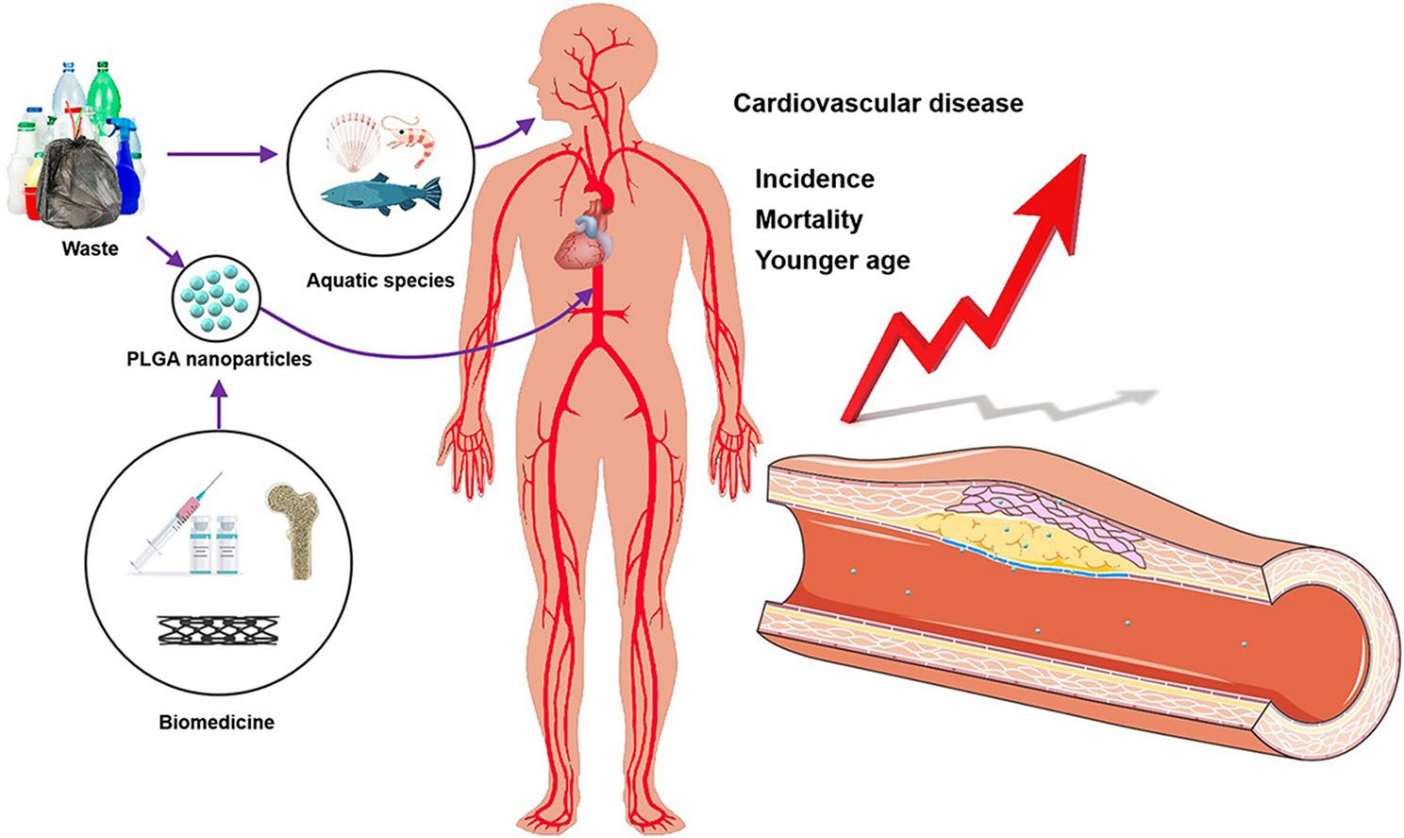

**Supplementary Information:**

The online version contains supplementary material available at 10.1186/s12951-023-01808-3.

## Background

Degradable materials have been expected as an alternative to plastic. Among them, biodegradable materials can be decomposed by enzymes or micro-organisms, and some can be degraded in the human body, finally form harmless products, which is considered to be environmentally friendly materials [1]. It has been widely used in catering products, packaging, automotive, consumer electronics, textiles, agriculture and other fields [2]. Plastics release micro- or nano-plastics (MNPs) along with the degradation process, which are washed away by water and preferentially enter aquatic organisms to cause damage [3]. MNPs damage the survival and reproduction of the organism, which can be detected in human blood [4, 5]. Biodegradable materials such as PLGA has been widely employed in medical therapy and diagnosis, therefore, the potential harm of degradable MNPs in the human body is attracting attention.

The impact of metal oxide nanoparticles on human health has always been concerned, it mainly leads to organic inflammation, genotoxicity and major cellular organelle dysfunction [6]. Cui et al. found both high dose and low dose of metal oxide nanoparticles caused severe cytotoxicity on metabolomics [7]. Sun et al. evaluated toxicity differences among varying size of SiO_2_ NPs on the cornea, which led to increased cell death and mitochondrial dysfunction in primary human corneal epithelial cells [8]. Kong found that Ni NPs had an apoptotic effect on Sertoli-germ cells of rats [9]. Furthermore, nickel (Ni) NPs injected intravenously in Sprague Dawley rats induced liver and spleen injury, lung inflammation, and caused cardiac toxicity [10, 11]. Toxicological studies on degradable biomaterials are limited, for instance Green et al. reported that in the salt-water organisms, like flat oyster *Ostrea edulis* and *Arenicola marina* L., high levels of poly lactic acid (PLA) in sandy sediments induce stress and elevate respiration rates [12, 13]. PLGA NPs were injected 0.5 mg under the skin of rats would have a probability caused pale kidneys and pyelectasis [14]. Thackaberry et al. reported that intravitreal administration of PLGA microspheres would cause a serious immune response of nonhuman primates and rabbits [15]. Grabowski et al. found PLGA NPs with negative charge induced higher cytokine secretions in human lung epithelial cells [16]. However, the current research cannot clarify the harmful effects of NPs of degradable materials on the circulation.

As a common cardiovascular disease, atherosclerosis (AS) is a main cause of death in the world. The incidence of vascular stenotic diseases represented by atherosclerosis increase by years, and the patients tend to be younger [17]. Fat streaks that form in blood vessels are the initial stage of the disease, which can happen as early as infants. For a long time before entering the advanced stage, they are in the incubation period and asymptomatic state [18]. Arterial stenosis is one of the typical pathological features of AS, and an important indicator of cardiovascular diseases risk. Furthermore, percutaneous coronary intervention also puts the patient at risk of developing in-stent stenosis, which is the slow renarrowing of a stented coronary artery lesion due to arterial injury, followed by the development of neointimal tissue [19, 20].

PLGA can be hydrolyzed into lactic acid and glycolic acid which can then be metabolized by humans [21]. Due to its biocompatibility and biodegradability properties, PLGA is certified by the US Food and Drug Administration (FDA) and the European Medicine Agency (EMA), which has been developed for use in nano-pesticides, food preservation, surgical sutures, bone and oral prosthetics, drug delivery, nanomedicine diagnostics, and intravascular stent [22, 23]. The industrial application of degradable materials such as PLGA also releases a large number of polymers into the environment. As it is mostly used in the biomedical field, it makes PLGA more accessible to the human body. Although degradable polymers are metabolically broken down after a period of time, before degradation, the polymers remain in the circulation in the form of nanometers. During this time, few studies on the impact of PLGA polymers on people have been published. The NPs were prone to accumulated in the main organs like liver, lung, spleen where they can be metabolized by macrophages [24, 25]. In previous studies, we reported that macrophages in the plaque phagocytized PLGA NPs accelerated the progression atherosclerosis in ApoE^−/−^ mice [26]. Also, the polymeric nanomicelles were considered to be a potential hazard for the cardiovascular disease [27]. Thus, taking the PLGA as an example, we study the effect of degradable material NPs on vascular stenosis in pre-atherosclerosis and vascular stenosis after interventional therapy.

In this research, we constructed an in vivo vascular stenosis model to investigate the effect of PLGA NPs on the stenosis progress [28, 29]. First, we prepared PLGA NPs and examined the influence of PLGA NPs on vessels with different degrees of stenosis, including intimal thickness, the distribution of smooth muscle cells (SMCs) and collagen. Next, since PLGA NPs directly contacted the vascular endothelium, we tested the effect of PLGA NPs on vascular endothelial function and vascular inflammation. To observe its role on endothelial cell phagocytosis and cell migration, we co-cultured vascular endothelial cells with PLGA NPs. Blood stream would produce abnormal shear stress at the branches and stenosis of blood vessel, which have been proven that it promoted AS [30]. To mimic the environment of disturbed flow in narrow locations, we treated endothelial cells with a mechanical loading device and found increased accumulation of PLGA NPs under disturbed flow [31, 32]. We revealed the potential risk of PLGA NPs in cardiovascular stenosis from the aspects on vascular inflammation and function. Moreover, PLGA NPs damaged endothelium under the disturbed blood flow at the stenosis site. This study can provide new perspectives for the design and application of polymeric nanomaterials in diagnosis and treatment. It reveals the potential cardiovascular risk of biodegradable PLGA polymer in our living environment on the human beings.

## Methods

### Materials

ApoE^−/−^ mice and C57 BL/6 mice (8 weeks, male) were purchased from Beijing Vital River Laboratory Animal Technology Co., Ltd. (Beijing, China). PLGA polymer powder: molecular weight 90,000, 50/50; the ingredients of High-fat diet (HFD) contained 20% protein, 40% carbohydrate, 40% lard and 0.15% cholesterol. The human umbilical vein endothelial cells (HUVECs) were purchased from ATCC cell bank (Manassas, VA, USA).

### Preparation and characterization of PLGA NPs

PLGA NPs were produced by nanoprecipitation process and labelled the PLGA NPs with DiI. Briefly, 50 mg PLGA was completely mixed into 5 mL dimethyl sulfoxide (DMSO) to obtain a PLGA stock solution. The PLGA mixture solution (5 mL) was taken and transferred into a 50 mL beaker. Adding 10 mL of distilled water drop by drop in the PLGA mixture with gentle stirring, after that PLGA mixture was dialyzed using dialysis bag (3.5 kDa, Solarbio, Beijing, China) to remove DMSO. The volume was replenished to 20 mL to obtain a concertration of 2.5 mg mL^−1^ PLGA NPs solution, stored at 4 °C. The protocols for the characterization of NPs were detailed in previous studies [26].

### Animal experiment

All animal procedures were approved by Laboratory Animal Welfare and Ethics Committee of Chongqing University for Animal Protection. Twenty ApoE^−/−^ male mice were randomly divided into five groups after an adaptive feeding week. Local left common carotid artery (LCCA) stenosis was created as manipulation with ~ 30% and ~ 70% stenosis estimation by calculation that have been determined by placing 9–0 nylon suture around the artery using an external blunt needle on middle or distal location of LCCA, which was removed subsequently [28]. We fixed mice by mouse tail vein injecting fixator (Zhenhuabio, China), and injected NPs at a dose of 10 mg kg^−1^ every two days to each group through tail vein. The control group was injected with 100 μL PBS. In the period of experiment, all the animals were fed with western diet, freely water, and the conditions of experimental animals were observed and recorded every day, which were lasted for 2 weeks.

After 2 weeks the mice were harvested. The blood was collected from the mice and preserved at 4 °C, after 6 h, centrifuged at 4000 rpm for 15 min to obtain serum. We measured the lipid profile using an automated biochemical analyzer (Shenzhen Redu Life Technology) according to previous studies [26]. We collected fresh vein blood from healthy C57 BL/6 mice. The experimental procedures for detecting hemolysis rate were carried out according to our previous studies [26].

### HE and IHC staining

Hematoxylin–eosin (HE) and Immunohistochemistry (IHC) staining of the carotid artery was performed as previously researches [26]. After dewaxing, MASSON, MOVAT, EVG, α-SMA, CD31, vWF, Ki67, P120 and Thrombomodulin (TM) staining were used to observe the vascular function, pathological feature and the distribution of collagen. Sections of the main organs were also analyzed by HE staining.

### HUVECs cell viability assay

HUVECs were cultured with medium containing 10% fetal bovine serum (FBS). At exponential growth stage, HUVECs were seeded in 96-well plates during phase log-growth. After 12 h, the medium was changed to serum-free medium. The cells were starved overnight, then treated with different concentrations (0, 50, 100, 200, 300 and 400 μg mL^−1^) of PLGA NPs. After incubating for 3, 6, and 12 h, MTS assay solution was added to each well and fetched out after 60 min. To mix colors, gently shake the culture plate for 15 s before testing. The OD value was measured at 490 nm by microplate reader (BioTek Instruments Inc., USA).

### PLGA NPs uptake by HUVECs

HUVECs were seeded into 24-well plate (contained glass coverslips) with a density of 2 × 10^5^ mL^−1^ and 10% FBS added into each well. After 12 h, the medium was changed to fresh medium containing 100 μg mL^−1^ of DiI@PLGA. HUVECs were treated for different time points (0.5, 2, 4, 8 and 12 h) to observe the PLGA NPs uptake. Then they were fixed and permeabilized. The nuclear were colored with DAPI. Each step was followed by washing with PBS three times. The cells on glass coverslips were observed and analyzed via confocal laser scanning microscopy.

### PLGA NPs influence to the VE-Cadherin

Monolayer HUVECs formed in 24-well plate were exposed to PLGA NPs. After 24, 48, and 72 h, the cells were fixed and permeabilized. Then the cells were blocked with blocking buffer for 1 h. HUVECs were incubated with VE-Cadherin (Santa Cruz, sc52751) and secondary antibody 1 h at room temperature separately. Then the same steps were repeated according to “PLGA NPs uptake by HUVECs” part.

### PLGA NPs influence to the HUVECs migration

HUVECs were seeded on a 6-well plate to 90% confluency, the scratches were made on the bottom of the 6-well plate. Then cells were treated with 100 μg mL^−1^ PLGA NPs for 24, 48 and 72 h and cultured with medium serum-free. Optical microscopy (Olympus Optical Co., Ltd.) was used to take photograph.

### Actin rearrangements induced by PLGA NPs exposure

The HUVECs were seeded into 6-well plate, after the cells adherence we put it on the horizontal platform of an orbital shaker (DS-5000, VWR International, West Chester, Penn) in the incubator for shaking 24 h and 72 h [31]. Each incubation time consisted of a control group and a group with PLGA NPs exposure. Then HUVECs were fixed and washed, for staining of the cell cytoskeleton, F-actin was used. Then the same steps were repeated according to “PLGA NPs uptake by HUVECs” part.

### Statistical analysis

All results were presented as mean ± standard deviation. For image data analysis and processing, Image J software was used and statistical procedures were performed using the Graphpad Prism 6. The significance of the variable of the detections and results were tested statistically by using the one-way ANOVA and Student’s t-test followed by Tukey’s Multiple Comparison Test. P < 0.05 was considered significantly.

## Results

### Preparation and characterization of PLGA NPs

**Fig. 1:**
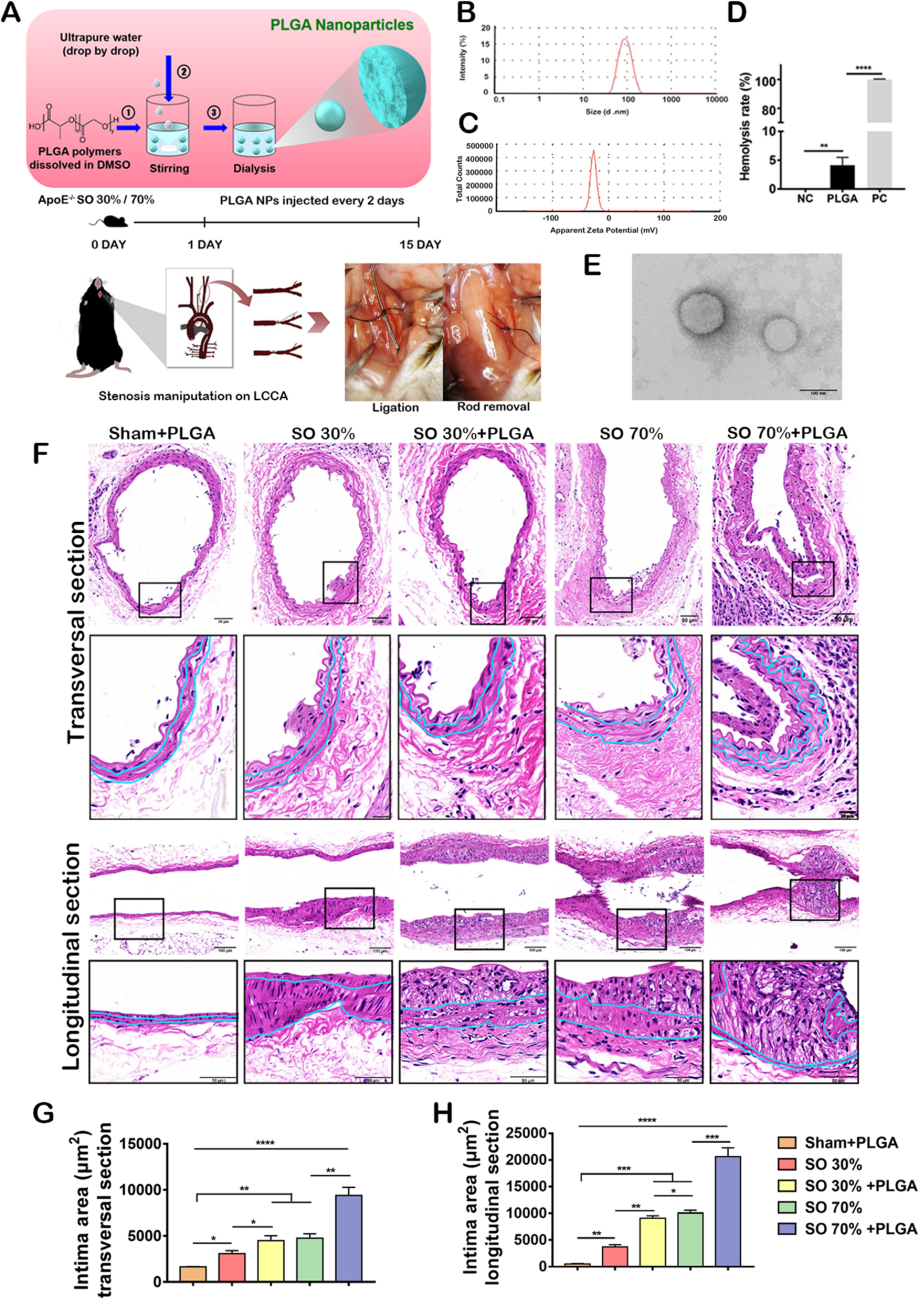
PLGA NPs preparation and administration accelerate intimal thicken after stenosis operation. **A** The illustration of PLGA NPs preparation (upper) and animal stenosis operation (lower). **B** PLGA nanoparticle size distribution (n = 3). **C** Measurement of PLGA NPs zeta potential (n = 3). **D** Hemolysis rate (%) of the nanoparticle dispersions (2 mg/mL), n = 3. **E** TEM image of PLGA NPs. Scale bars, 100 nm. **F** Transversal and longitudinal sections of the HE staining of ApoE^−/−^ mice carotid artery, which showed the changes in the structure of vessel walls after NPs treatment and SO. Scale bars, upper, 50 μm, lower, 20 μm. Quantitative analysis of intimal area of carotid artery in transversal section (**G**) and longitudinal section (**H**). Scale bars, upper, 100 μm, lower, 50 μm, n = 5, *p < 0.05; **p < 0.01; ***p < 0.001; ****p < 0.0001

To start with, PLGA NPs were prepared by nanoprecipitation (Fig. 1A). Then, the characterization of the as-prepared PLGA NPs was carried out by a dynamic light scattering (DLS) experiment, resulting in an average size of 85.7 ± 3.09 nm with a zeta potential of -26.6 ± 1.0 mV. The result proved that the NPs were in the normal nano size and properties (Fig. 1B, C). It has been proved that when the particles are smaller than 100 nm, they could be easily taken up by the tissue. Besides, the result of the transmission electron microscope (TEM) showed that NPs had a regular round shape and a similar size to the previously reported data (Fig. 1D) [26]. It also showed that the NPs were well distributed in an aqueous solution with an appropriate hemolysis rate (< 5%) (Fig. 1D, E). In brief, we successfully prepared PLGA NPs and tested the physicochemical properties of the NPs.

### PLGA NPs accelerate stenosis progression

A stenosis operation (SO) experiment was designed to investigate the influence of PLGA NPs on the progression of arterial stenosis. At first, the LCCA of a 12-week-old ApoE^−/−^ mouse was partially ligated (shown in Fig. 1A). Then, a short rod was tied to the LCCA with sutures. The rod had a diameter of 30% and 70% of the average size of the mouse carotid artery, respectively. After the short rod was removed, the local area of the blood vessel lost 70% and 30% correspondingly, resulting in stenosis. Next, the mice were on an HFD, followed by several injections of PLGA NPs every other day for two weeks. As shown in the HE staining results of the transversal and longitudinal sections of the blood vessel (Fig. 1F), the SO induced a corresponding reduction in the lumen area, suggesting that the SO model was feasible for further study. Compared to only the 30% SO group, the lumen area in the corresponding group treated with PLGA became smaller. Besides, the 70% SO groups treated with/without PLGA NPs showed similar results, which demonstrated that the NPs promoted the progress of stenosis. At the same time, the microscopic analysis showed that the morphology of the vascular wall had changed greatly by NPs. According to Fig. 1G and H, the intima area was increased by only SO with HFD. However, PLGA NPs exacerbated this effect. The media area and adventitia area of the vascular wall were also affected by SO and PLGA NPs injection, although there was no distinct statistical difference (Additional file 1: Fig. S1A–D). We calculated the inner diameter of the vessels in transversal section and longitudinal section (Additional file 1: Fig. S1E and F). When treated with PLGA NPs, the inner diameter would decrease significantly both in 30% stenosis and 70% stenosis group. In short, PLGA NPs promoted intimal hyperplasia in the stenosis and led to the decrease of the lumen area.

### Extracellular matrix are deposited in the stenosis vascular wall accompanied with the PLGA NPs administration

**Fig. 2:**
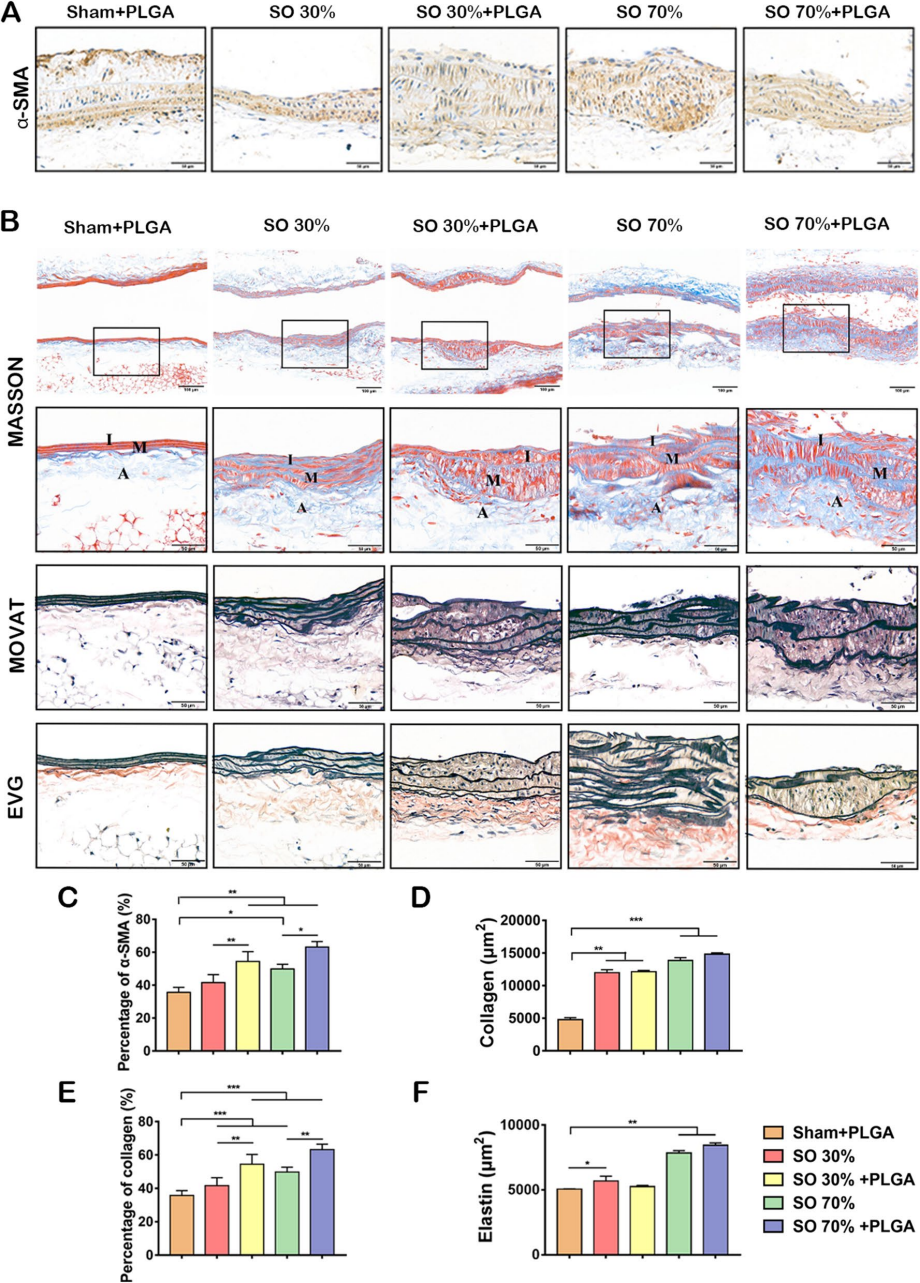
The distribution of VSMCs and ECM in blood vessel wall is changed by stenosis operation and NPs administration in the stenosis area. **A** IHC staining for α-SMA of carotid artery longitudinal sections after stenosis operation and PLGA NPs treatment (n = 4, scale bars, 50 µm). **B** Masson’s trichrome (MASSON) staining (upper), collagen fiber (red), muscle fiber (blue); MOVAT staining (middle), collagen fiber (black), muscle fiber (red); EVG Staining of carotid artery longitudinal sections (lower) (scale bars, 500 µm; 50 µm); elastic fiber (black/purple); muscle fiber (red); A: Adventitia; M: Media; and I: Intima; SO: stenosis operation. **C**The quantification of α-SMA expression (n = 4). Quantification of **D** collagen of MASSON staining, **E** collagen percentage of MOVAT staining and **F** elastin integrity of EVG staining, *p < 0.05; **p < 0.01; ***p < 0.001 (n = 5)

It has been known that for the purpose of vessel wall healing, vascular smooth muscle cells (VSMCs) tend to become proliferative in response to injury. Excessive proliferation and migration of VSMCs from the media into the intima, however, results in the formation of neointima and vascular occlusion [33]. Based on that, the effect of PLGA NPs on the VSMCs of the blood vessels in the stenosis was tested. As shown in Fig. 2A, we observed the alpha-smooth muscle actin (α-SMA) that is a marker of VSMCs was expressed in both media and neointima. The percentage of α-SMA are enhanced after 70%SO, indicating that VSMCs were proliferated. After PLGA injection, the corresponding areas further expanded, and the intima became thicker (Fig. 1F–H, Fig. 2C). The inner diameter of blood vessels has decreased after treated with PLGA NPs in SO group, though no significant difference between SO 30% and SO 30% + PLGA group (Additional file 1: Fig. S1E and F). These results demonstrated that PLGA NPs would enhance the proliferation of VSMCs and induced diffuse intimal thickening in the narrow area. Then, the MASSON, EVG, and MOVAT staining were used to observe the distribution of extracellular matrix (ECM) in the stenosis sites, such as collagen fibers, elastic fibers, and muscle fibers (Fig. 2B). In the sham group, the collagen fibers were thin and slightly curved. However, after SO, the blood vessel wall was thickened, the elastic plate was bent irregularly, and collagen fibers were deposited. Moreover, with SO and PLGA NPs injection, the blood vessel wall was even thicker, while collagen fiber deposition further increased and fractures appeared. Intimal hyperplasia occurred in the 70% SO group and 70% SO + PLGA group. In general, SO together with PLGA NPs injection had thicker blood vessel walls, worse fiber integrity, and more severe intimal hyperplasia than SO alone (Fig. 2D–F). Thus, it showed that the injection of PLGA NPs accelerated the proliferation of VSMCs and tended to break the collagen and elastic fibers in the blood vessel wall.

### PLGA NPs decrease the endothelium function of blood vessels in stenosis sites

**Fig. 3:**
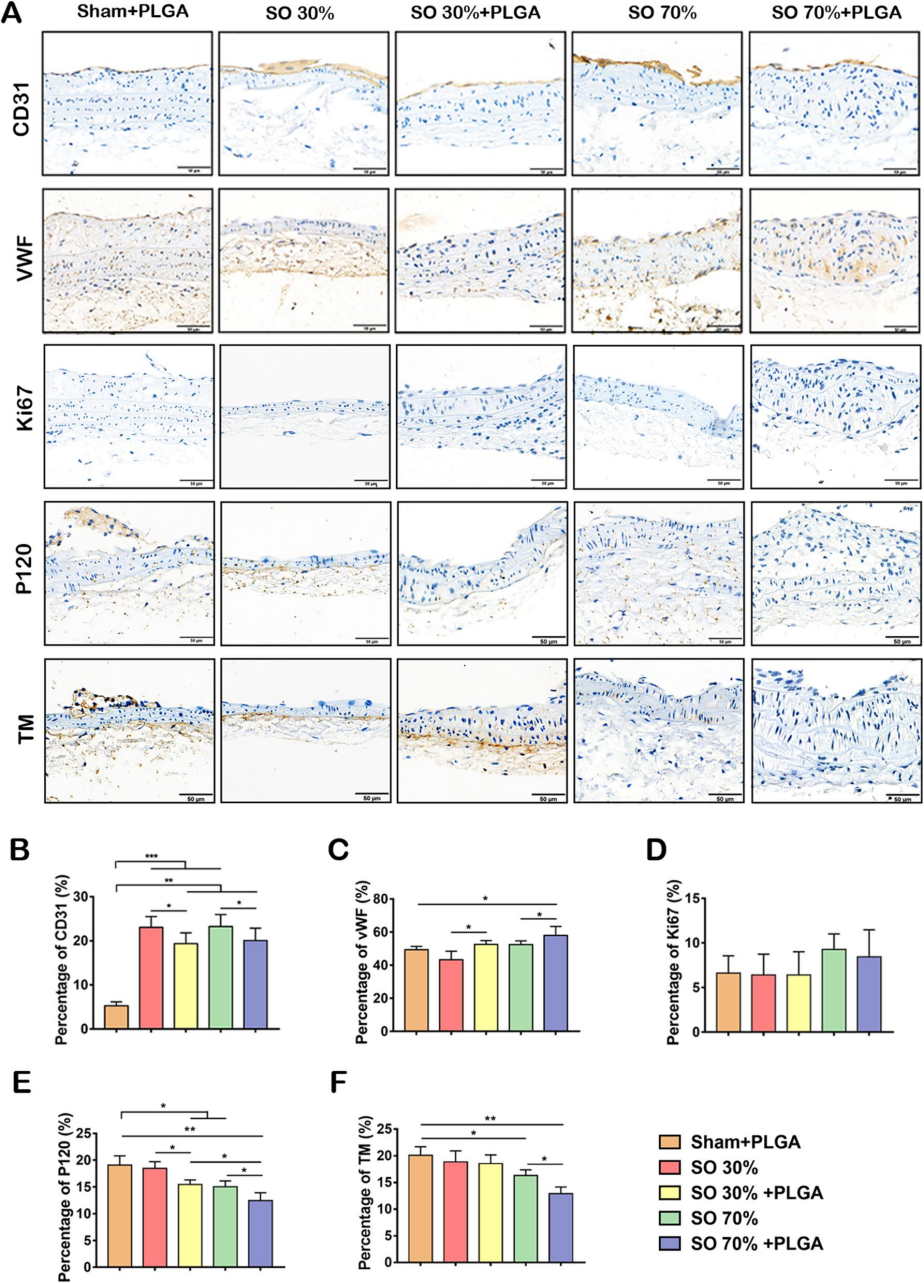
PLGA NPs administration influence the function of vascular wall in stenosis area. **A** IHC staining for CD31, vWF, Ki67, p120 and TM of carotid artery longitudinal sections. Scale bars, 50 µm. **B**–**G** And the quantification of percentage of CD31 expression (**B**); percentage of vWF positive expression (**C**); percentage of Ki67 positive expression (**D**); percentage of p120 positive expression (**E**); percentage of TM positive expression (**F**) in different groups, *p < 0.05; **p < 0.01; ***p < 0.001 (n = 5)

To evaluate the effect of SO with/without PLGA NPs treatment on the endothelium function of blood vessels, a panel of markers of endothelial function was profiled using IHC (Fig. 3A). The expression of CD31 was used to indicate the re-endothelialization in injured vessels as described in the previous study was investigated [34, 35]. In the SO group without PLGA NPs injection, the CD31 expression was higher than in other groups. It indicated that the increase of CD31 expression enhanced re-endothelialization and endothelial proliferation in response to injured vessels. Interestingly, the SO combined with the PLGA NPs group had a lower CD31 expression level compared to the group with no PLGA NPs injection. It suggested that PLGA NPs could decrease the CD31 expression and might lead to endothelium dysfunction (Fig. 3B). Only megakaryocytes and endothelial cells (ECs) generate Von Willebrand factor (vWF), which is a large multimeric glycoprotein. By mediating platelet adherence to active and damaged vessels, it aids in hemostasis and thrombosis [36]. In addition, inflammation- induced vWF secretion can enhance the interaction between vWF and platelets, promoting thrombosis [37]. As a result, the expression of vWF was increased after PLGA NPs treatment, suggesting the promotion of the stenosis inflammation (Fig. 3C). However, the Ki67 expression, which indicate the endothelial proliferation, did not change significantly between the groups (Fig. 3D). Furthermore, the effect of PLGA NPs on cell adhesion and atherosclerotic thrombosis was also investigated. The p120 expression is normally related to the VE-Cadherin in ECs [38]. Additionally, thrombomodulin (TM), which is related to the occurrence of thrombosis, is expressed in normal vascular arteries [37]. It was found that intercellular adhesion of blood vessels at stenosis was decreased after PLGA NPs treatment. The expression of TM was decreased demonstrating an increased risk of thrombosis (Fig. 3E, F). Overall, with the treatment of PLGA NPs, the secretion of vWF in the stenosis area increased, endothelial cell function was dysregulated, intercellular adhesion and the expression of TM was decreased. As a result, these effects on blood vessels would boost the risk of AS.

### PLGA NPs accumulate in stenosis sites and promote the secretion of inflammatory factors

**Fig. 4:**
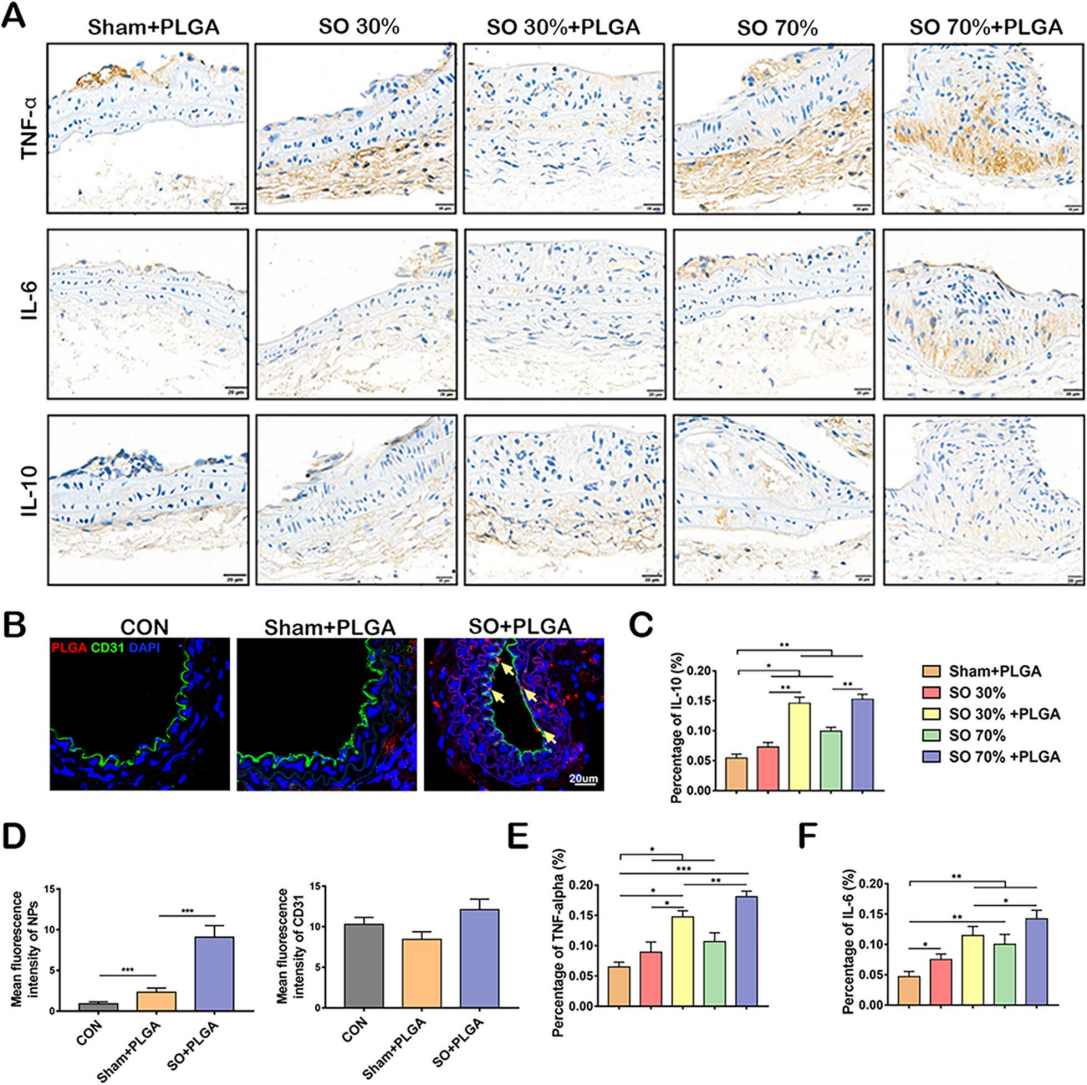
PLGA NPs deposition at stenosis sites promote inflammatory factors secretion. **A** IHC staining for TNF- α, IL-6, and IL-10 (scale bars, 20 µm). **B** Comparison of deposition of NPs in blood vessels. And the quantification of percentage of IL-10 positive expression (**C**); percentage of TNFα positive expression (**E**) and percentage of IL-6 positive expression (**F**) in different groups. **D** The quantification of NPs and CD31. *p < 0.05; **p < 0.01; ***p < 0.001 (n = 5)

Inflammation can cause AS by changing the function of the cells in the arterial wall [39]. At the beginning of the atherosclerotic process, pro-inflammatory cytokines such as interleukin-6 (IL-6) and tumor necrosis factor-α (TNF-α) are released, followed by the anti-inflammatory cytokine interleukin-10 (IL-10) [40, 41]. The TNF-α and IL-6 expression levels in blood vessels were measured in this investigation. The up-regulation of inflammatory factors was discovered to be triggered by SO, and the expression of inflammatory factors was directly proportional to the degree of SO. At the same time, the SO + PLGA NPs group showed an enhanced expression of inflammatory factors compared with the SO group. Also, the expression of anti-inflammatory factor IL-10 in blood vessels was tested, showing similar trends (Fig. 4A, C–E). These results proved that PLGA NPs could promote vascular inflammation, inferring that the treatment of NPs could influence the ECs and VSMCs. It was also found that the deposition of PLGA NPs in the site of vascular stenosis was increased in comparison to a control group. After NPs injection, a lot of NPs had entered blood vessels through ECs in the SO + PLGA NPs group after 24 h, indicating that abnormal shear stress induced by vascular stenosis promoted the deposition of NPs. (Fig. 4B, F, G).

### PLGA NPs are phagocytized by ECs decreasing cell viability and cell migration

**Fig. 5:**
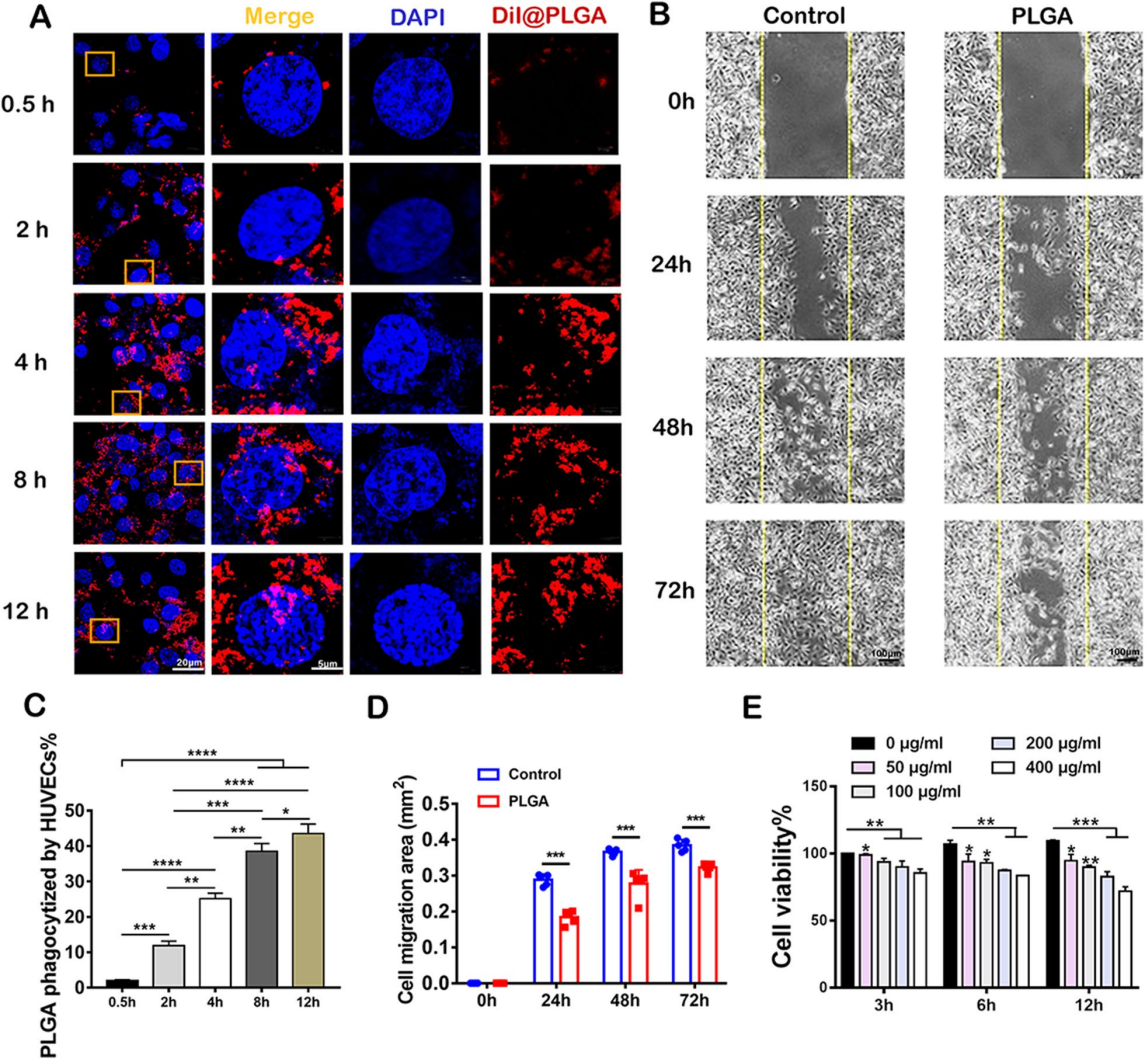
PLGA NPs are phagocytized by ECs in time-dependent manner, decreasing cell viability and cell migration. **A** HUVECs uptake of PLGA NPs on 0.5, 2, 4, 8, and 12 h. Scale bars, left, 20 µm, right, 5 µm. **B** The HUVECs migration morphology influenced by PLGA NPs. Scale bar, 100 µm. **C** The quantification of PLGA NPs uptake by HUVECs (n = 5). **D** Cell migration area of HUVECs influenced by PLGA NPs (n = 5). **E** Different concentrations of PLGA NPs and time points impact on the viability of HUVECs. (n = 3) *p < 0.05; **p < 0.01; ***p < 0.001

ECs are located in the inner lining of vascular walls. They’re also vital for preserving and absorbing cell detritus and foreign materials, which makes it a crucial concern when evaluating the safety of PLGA NPs. At 0.5 h, the percentage of PLGA NPs uptake by HUVECs was 2.036 ± 0.252% (Fig. 5A and C). At 2 h, it increased greatly by 5 folds compared to that of 0.5 h incubation. With a longer incubation time, the uptake of PLGA NPs consistently showed a higher percentage. Furthermore, the stability of ECs is important during injury, especially when foreign materials like NPs try to enter and affect the stability of the ECs. According to the experimental results, the PLGA NPs could inhibit cell migration over time (Fig. 5B and D). According to ISO guidelines for the biological evaluation of medical devices, non-toxic or mildly cytotoxic materials are clinically acceptable. In a previous study, up to a concentration of 300 µg mL^−1^ of PLGA NPs, no substantial fatal toxicity was observed [42]. Herein, the cell viability of HUVECs was tested at high concentrations (assuming the cytotoxic concentrations: 50, 100, 200, 300, and 400 µg mL^−1^) which was higher than 70–80% of the control group (Fig. 5E). The viability of HUVECs was considerably influenced by PLGA NPs at higher concentrations. At the highest concentration, the percentage of cell viability was even higher than 70% as previously reported. Conclusively, PLGA NPs phagocytosis by ECs was positively correlated with incubation time. PLGA NPs decreased the progress of endothelial cell migration. The high concentration of PLGA NPs influenced ECs viability in a time- and dose-dependent manner, even if the viability of HUVECs remains above 70%.

### PLGA NPs induce endothelial leakiness

**Fig. 6:**
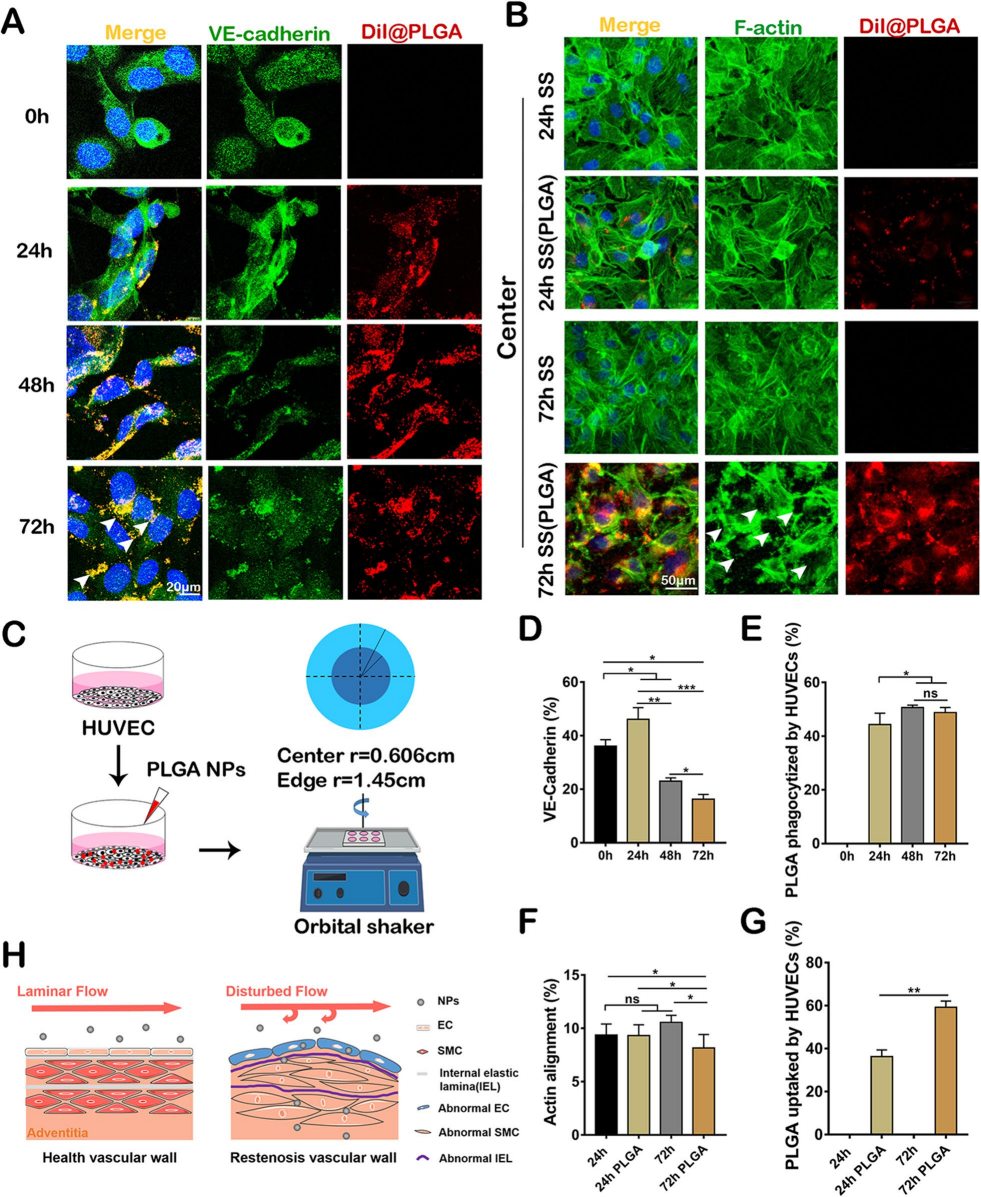
PLGA NPs can impair the function of vascular ECs. **A** The expression of VE-cadherin and NPs uptake of HUVEC in 24, 48, and 72 h. Scale bar, 20 µm. **B** Cytoskeleton and NPs uptake of HUVEC under low shear stress in 24 and 72 h. Scale bar, 50 µm. **C** Schematic illustration of a mechanical experimental device by mimicking shear stress using an orbital shaker of a 6-well plate and approximate low shear stress range in the center area. **D**, **E** The quantification analysis of VE-Cadherin and PLGA NPs uptake on HUVECs (n = 5). **F**, **G** Changes of fractal dimension in cytoskeleton and PLGA NPs uptake of HUVECs under low shear stress (n = 5), *p < 0.05; **p < 0.01; ***p < 0.001; ****p < 0.0001. **H** Illustration of the negative effect of PLGA NPs on the site of stenosis

To determine VE-Cadherin expression of the ECs after the incubation with PLGA NPs, experiments for VE-Cadherin detection were carried out using 100 µg mL^−1^ NPs with different incubation periods (0, 24, 48, and 72 h). As a result, the percentage of VE-Cadherin expression increased to 46.127 ± 5.234% at 24 h. However, VE-Cadherin expression decreased gradually at 48 and 72 h with the exposure to PLGA NPs, resulting in 23.017 ± 1.461% and 16.309 ± 2.291% (P < 0.05), respectively (Fig. 6A, D and E). For the detection of endothelial cell phagocytosis induced by PLGA NPs, more than 40% of ECs had swallowed NPs at 24 h. With the time increasing, the number of ECs that swallowed NPs had a certain increase at 48 and 72 h. However, there was no statistical difference in the percentage of ECs that swallowed PLGA NPs between 48 and 72 h, which indicated that the NPs phagocytosis of ECs reached a saturated state. These results suggested that the PLGA NPs might cause damage to the endothelial barrier, thus promoting phagocytosis. To investigate whether the exposure to PLGA NPs dysregulated endothelial barrier by altering cytoskeletal structure, a previously reported mechanical loading model was applied in this study [31]. The orbital shaker was subjected to low shear stress loading in the middle of the six-well plate at 150 rpm, mimicking the mechanical conditions at the plaque of AS and vascular stenosis. HUVECs were exposed to low shear stress and NPs for 24 and 72 h. The fractal dimension in the actin cytoskeleton was then analyzed with Image J (Fig. 6C). Actin rearrangement in response to the shear stress exposure was observed in HUVECs treated with PLGA NPs (Fig. 6B and F) at different incubation times. Cytoskeletal of HUVECs was immuno-stained, and the protein expression and distribution in ECs were analyzed. The quantification of actin alignment which was presented by fractal dimension did not show a significant difference within the control group. However, fractal dimension in PLGA NPs with 72 h incubation time showed a significant difference and higher than others (P < 0.05), which proved that those NPs could destroy cytoskeletal under low shear stress after a long time. At 24 h, even the PLGA NPs group showed no significant difference of statistical analysis in fractal dimension compared to the control group. Moreover, the quantification of PLGA NPs uptake by HUVECs (Fig. 6G) (P < 0.01) represented a significant difference between 24 and 72 h incubation time.

According to the above description, PLGA NPs might disturb the endothelial barrier of HUVECs, which led to endothelial dysfunction. Then, the down-regulated expression of VE-cadherin followed by increased ECs engulfment of PLGA NPs interfered with the integrity of the endothelial barrier and contributed to the endothelial leakiness.

The effects of SO and PLGA NPs administration on plasma lipid levels were also investigated. The TC, TG, and HDL-C test showed a substantial distinction (P < 0.05) in the manipulated stenosis group accompany by PLGA NPs injection compared to the group with no PLGA NPs treatment (Additional file 1: Fig. S2A). However, this significant difference did not always occur in the same manipulated stenosis setting level in each test, which suggested that it did not represent a definite trend line. Thus, the results revealed that the formation of stenosis would not cause obvious changes in plasma lipids. According to morphological images, there were no aberrant changes in the primary organs of mice in all groups (Additional file 1: Fig. S2B). It was evidenced that PLGA NPs did not influence the plasma lipids and they had no toxicity in the main organs.

It can be concluded that the PLGA NPs could be more easily phagocytized by ECs in the disturbed flow area and could enter the interior of the vessel wall, which would induce ECs dysfunction, increased permeability, and further promote the accumulation of PLGA NPs. It led to inflammation and abnormal distribution of internal elastic lamina, affecting the physiological activity of SMCs. Pathologically, PLGA NPs could accelerate the progress of stenosis. (Fig. 6H).

## Discussion

With the potential harm of NPs to the human body, the cardiovascular system has been recognized as one of the targets of nanotoxicity. We have explored the PLGA NPs effects on the cardiovascular diseases, stenosis progression, and possible related mechanisms. For detecting the effect of NPs on the progress of vascular stenosis, we performed surgery for focal stenosis of the left carotid artery in ApoE^−/−^ mice. This method could reduce the luminal area and induce ECM deposition (Figs. 1F–H, 2B, D–F). Consequently, neointimal hyperplasia with distinct morphologic characteristics in the artery wall was found after two weeks, which indicating stenosis. The model was developed based on earlier techniques that LCCA blood flow was disrupted in mice by total ligation or flow limitation via outflow branch ligation [29]. To investigate the effects of NPs on the progression of stenosis and atherosclerotic lesions, the ApoE^−/−^ mice were performed in two degrees of stenosis: ~ 30% and ~ 70% in LCCA, then were fed HFD and injected with PLGA NPs. We found that plaques would be formed after carotid stenosis surgery two weeks. We focused on stenosis and the pathological changes of early AS. In order to discuss endothelial function clearly, we expect to reduce the impact of plaques.

We found that the involvement of HFD and PLGA NPs promoted stenosis development in the vessel walls. In the narrow site after the NPs injection, factors that have been shown to promote stenosis were observed, including the increased proliferation of VSMCs, endothelial damage, thrombosis risk, inflammation, changes in collagen distribution, and accelerated deposition of PLGA NPs. With the increased uptake of PLGA NPs by HUVECs, endothelial permeability and cell migration ability decreased, proving that the PLGA NPs might cause endothelial dysfunction. Under shear stress, PLGA NPs were found to affect actin rearrangement. Lactic and glycolic acids are PLGA degradation products that can enter the citric acid cycle and be metabolized, which might increase vascular inflammation. Chen et al. reported that the lactic acid generated by the PLLA degradation process could induce human aortic endothelial cells inflammation [43]. Therefore, we inferred that the polymers with similar compositions would have a similar effect.

PLGA NPs absorbed the protein in serum formed NPs with protein corona. Also, the degradation process of nanometer and its surface charge would affect the biophysical characteristics. Our previous research detected the physicochemical properties and morphology of PLGA NPs with protein corona, meanwhile, PLGA NPs with protein corona promoted the formation of foam cells was found [26]. The chemical composition of PLGA NPs was detected in previous work [44]. Meanwhile, we considered that the application research on carbon nanotubes and CuS NPs is popular, so the safety assessment of these materials would be challenging [45–47]. Different ratios of lactic acid and glycolic acid have different degradation rates of PLGA. PLGA (50:50) has the fastest degradation rate in the same molecular weight [48, 49]. It takes 8 weeks for PLGA (50:50) to be completely hydrolyzed and can be retained in the human body for a period of time [50]. It had reported that PLA/PLGA could accelerate the degradation rates under weakly acidic condition [51]. Zeng et al. found the 50:50 PLGA NPs acidified the solution at a faster rate than 75:25 PLGA NPs and PLA NPs [52]. PLGA NPs with negative charge are more likely to be phagocytized, which can be conducive to observation [53]. Furthermore, PLGA usually needs PEG modification or biomimetic membrane coating in practical applications in order to prolong the circulation time. Therefore, the harm of polymers such as PLGA to the human body needs further investigation.

The result of cell viability revealed the relative viability of PLGA group was higher than 70–80% of the control group. It is considered as non-toxic or slight cytotoxic and clinically acceptable according to the guidance of ISO 10993-5 for the biological evaluation of medical devices, and in previous study has been explored up to a concentration of 300 μg mL^−1^ PLGA NPs did not cause significant lethal toxicity [42]. Considering the weight and blood content of mice, we designed the dose of 10 mg kg^−1^ for treatment, the average NPs concentration in each mouse would lower than 300 μg mL^−1^. Therefore, this reflects the PLGA NPs could impair blood vessels despite at the safe dosage. In clinical, PLGA is the form of microparticle made for drug loading. Park et al. summarized the PLGA-based injectable depot formulations clinically, which have introduced the dose of each PLGA-based microparticle [54, 55]. The research of the biomedical application of PLGA is almost nanoscale, but these studies are mainly conducted on small rodents. The dose of PLGA NPs used in our research helps to provide safety evaluation for future drug design.

ECs in blood vessels are the first barrier to contact NPs in blood circulation. However, the effect of ECs that have phagocytized NPs on VSMCs is worthy of further consideration. At the same time, the mechanism of NPs on VSMCs after entering the media is also interesting. In this study, the distribution of collagen in the stenosis model and the effect of NPs on VSMCs were examined. It was found that the area of collagen was increased. Collagen is mainly synthesized by VSMCs in vessels. The result indicated that NPs could stimulate VSMCs to secrete the amount of collagen in the stenosis area, suggesting the transfer of VSMCs from contractile to synthetic VSMCs. In addition, the NPs promoted the expression of α-SMA in the stenosis, showing the proliferation of VSMCs under the stimulation of the NPs, which was in line with the past investigations [56]. However, the mechanism of VSMCs interplaying with ECs which were impaired by NPs under abnormal shear stress still need to be studied.

It is worth noting that in the arterial circulation, abnormal hemodynamics not only exists in vascular stenosis and plaque areas, but also appears in the branches and bends of the aorta. The effect of NPs on these parts in the application of diagnosis and treatment has been rarely studied, which is of significance to investigate.

## Conclusions

We studied on the negative effects and mechanism of the accumulation of biodegradable PLGA NPs in the cardiovascular system, especially in the area of abnormal hemodynamics. In the area of disturbed flow, PLGA NPs were prone to be engulfed by ECs and deposited in vascular wall, which led to abnormal distribution of ECM, promoted the proliferation of VSMCs. We found that PLGA NPs were more likely to aggregate at area of stenosis, reduced endothelial function and promoted inflammation, which aggravated the process of stenosis. In addition, we proved PLGA NPs influence to the endothelial cell leakiness leads to endothelial cell dysfunction in vitro. PLGA NPs enhanced the endothelial permeability and reduced cell migration ability, and affected actin rearrangement under shear stress application which prove that PLGA NPs might cause endothelial dysfunction. The neglected hazard for polymeric NPs and potential risk in cardiovascular stenosis were revealed. The PLGA NPs were found to accumulate in the stenosis area, inducing endothelium dysfunction and inflammation, thus increasing the vascular stenosis. The application of biodegradable polymeric materials in our living environment especially in the cardiovascular field is proposed to require more in-depth and detailed research on safety and evaluation.

## Supplementary Information

**Additional file 1: Figure S1.** The quantitative of ApoE^-/-^ mice carotid artery HE staining longitudinal and transversal section. Quantitative analysis of media area (A) and adventitia area (B) in carotid artery longitudinal section. The quantitative analysis of media area (C) and adventitia area (D) in carotid artery transversal section. (E) Quantitative analysis of inner diameter in carotid artery longitudinal section. (F) Quantitative analysis of inner diameter in carotid artery transversal section. n= 5 , *p < 0.05; **p < 0.01; ***p < 0.001; ****p < 0.0001. **Figure S2.** Biosafety of PLGA NPs administration and restenosis manipulation. (A) The plasma lipid and lipoprotein levels were measured in Sham group, SO 30 % and SO 70 % with or without NPs treatment (n = 5), *p < 0.05. From top to bottom are triglycerides, total cholesterol, HDL-C, LDL-C. (B) The HE staining of main organs from different groups were representative. Scale bars, 50 μm.

## Data Availability

All data generated or analyzed during this study are included in this published article and its additional information files.

## Abbreviations

PLGA: Poly (lactic-co-glycolic acid)
NPs: Nanoparticles
MNPs: Micro- or nano-plastics
PLA: Poly lactic acid
AS: Atherosclerosis
FDA: Food and drug administration
EMA: European medicine agency
SMCs: Smooth muscle cells
HFD: High-fat diet
HUVECs: Human umbilical vein endothelial cells
DMSO: Dimethyl sulfoxide
LCCA: Local left common carotid artery
HE: Hematoxylin–eosin
IHC: Immunohistochemistry
TM: Thrombomodulin
FBS: Fetal bovine serum
DLS: Dynamic light scattering
TEM: Transmission electron microscope
SO: Stenosis operation
VSMCs: Vascular smooth muscle cells
α-SMA: Alpha-smooth muscle actin
ECM: Extracellular matrix
ECs: Endothelial cells
vWF: von willebrand factor
IL-6: Interleukin-6
TNF-α: Tumor necrosis factor-αlpha
IL-10: Interleukin-10
