# Correction: Biodegradable polymeric nanoparticles increase risk of cardiovascular diseases by inducing endothelium dysfunction and inflammation

**DOI:** 10.1186/s12951-023-01839-w

**Published:** 2023-03-16

**Authors:** Wen Shi, Atik Rohmana Maftuhatul Fuad, Yanhong Li, Yang Wang, Junyang Huang, Ruolin Du, Guixue Wang, Yazhou Wang, Tieying Yin

**Affiliations:** 1 Key Laboratory for Biorheological Science and Technology of Ministry of Education, State and Local Joint Engineering Laboratory for Vascular Implants, Bioengineering College of Chongqing University, Chongqing, 400044 China; 2 School of Medicine, Chongqing University, Chongqing, 400030 China

**Correction: Journal of Nanobiotechnology (2023) 21:65** **https://doi.org/10.1186/s12951-023-01808-3**

Following publication of the original article [1], the authors would like to update the funding note. The correct version of funding note is given in this correction.

This research was supported by the National Natural Science Foundation of China (12032007, 32171334, **12272071**) and the Key and Postdoctoral Projects of Natural Science Foundation of Chongqing (cstc2019jcyj-zdxmX0009 and cstc2020jcyj-bsh0139).

The original article has been corrected.

## References

[1] Shi W, Fuad ARM, Li Y, Wang Y, Huang J, Du R, Wang G, Wang Y, Yin T (2023). Biodegradable polymeric nanoparticles increase risk of cardiovascular diseases by inducing endothelium dysfunction and inflammation. J Nanobiotechnol.

