# Supplementary material for: Biodegradable polymeric nanoparticles increase risk of cardiovascular diseases by inducing endothelium dysfunction and inflammation
Source: J Nanobiotechnology. 2023 Feb 24;21:65. doi: 10.1186/s12951-023-01808-3 (PMC9951517; doi:10.1186/s12951-023-01808-3)
Supplement: Supplementary file 1 — Additional file 1: Figure S1. The quantitative of ApoE-/- mice carotid artery HE staining longitudinal and transversal section. Quantitative analysis of media area (A) and adventitia area (B) in carotid artery longitudinal section. The quantitative analysis of media area (C) and adventitia area (D) in carotid artery transversal section. (E) Quantitative analysis of inner diameter in carotid artery longitudinal section. (F) Quantitative analysis of inner diameter in carotid artery transversal section. n= 5 , *p < 0.05; **p < 0.01; ***p < 0.001; ****p < 0.0001. Figure S2. Biosafety of PLGA NPs administration and restenosis manipulation. (A) The plasma lipid and lipoprotein levels were measured in Sham group, SO 30 % and SO 70 % with or without NPs treatment (n = 5), *p < 0.05. From top to bottom are triglycerides, total cholesterol, HDL-C, LDL-C. (B) The HE staining of main organs from different groups were representative. Scale bars, 50 μm. [file 12951_2023_1808_MOESM1_ESM.pdf]

Additional file

Biodegradable polymeric nanoparticles increase risk of cardiovascular diseases by inducing endothelium dysfunction and inflammation

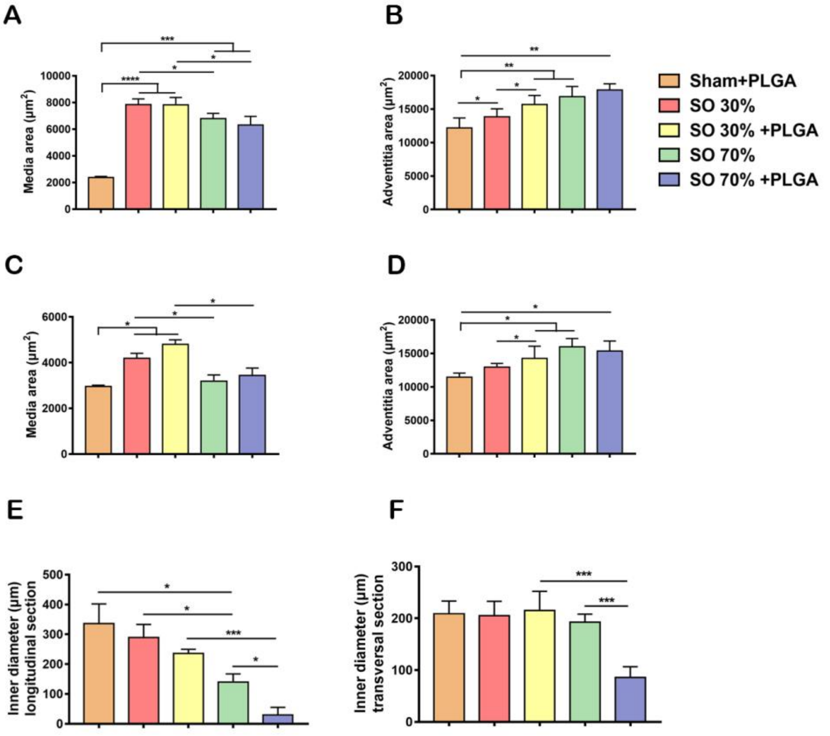

Figure S1 The quantitative of ApoE$^{-/-}$ mice carotid artery HE staining longitudinal and transversal section. Quantitative analysis of media area (A) and adventitia area (B) in carotid artery longitudinal section. The quantitative analysis of media area (C) and adventitia area (D) in carotid artery transversal section. (E) Quantitative analysis of inner diameter in carotid artery longitudinal section. (F) Quantitative analysis of inner diameter in carotid artery transversal section. n=5, *$p < 0.05$; **$p < 0.01$; ***$p < 0.001$; ****$p < 0.0001$.

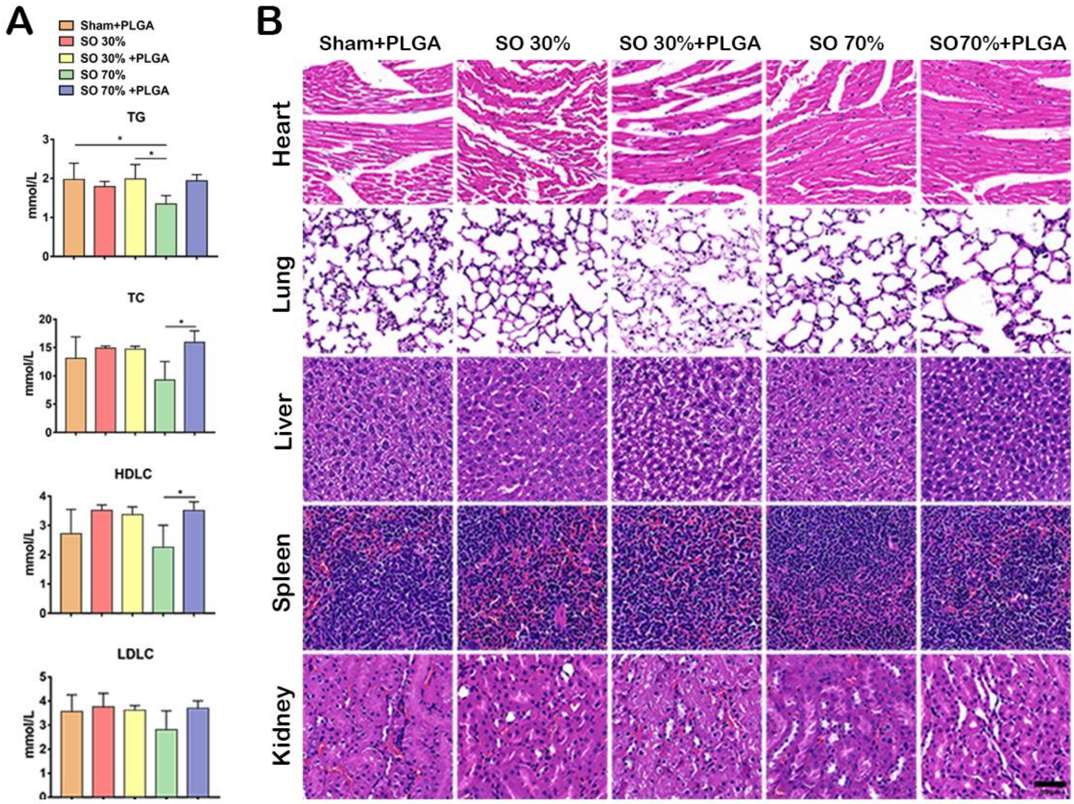

Figure S2 Biosafety of PLGA NPs administration and restenosis manipulation. (A) The plasma lipid and lipoprotein levels were measured in Sham group, SO 30% and SO 70% with or without NPs treatment ($n = 5$), $*p < 0.05$. From top to bottom are triglycerides, total cholesterol, HDL-C, LDL-C. (B) The HE staining of main organs from different groups were representative. Scale bars, 50μm.
